# Gut dysbiosis, metabolic signals, and pulmonary immune reprogramming: decoding the gut microbiota -immune axis in stroke-associated pneumonia

**DOI:** 10.3389/fimmu.2026.1812306

**Published:** 2026-08-27

**Authors:** Li Yang, Jingru Han, Shengqiang Li, Xinyu Wang, Yujie Jia, Zhijun Bao, Ting Ge, Guixin He, Juyue Zhou, Jiaming Cui, Yuanyuan Tian, Yingying Xie, Lefan Liu, Jinghui Du, Wentao Li, Jianchun Yu

**Affiliations:** 1 Department of Central Laboratory, First Teaching Hospital of Tianjin University of Traditional Chinese Medicine, Tianjin, China; 2 National Clinical Research Center for Chinese Medicine, Tianjin, China; 3 Department of Graduate School, Tianjin University of Traditional Chinese Medicine, Tianjin, China; 4 Department of Traditional Chinese Medicine, Tianjin University of Traditional Chinese Medicine, Tianjin, China; 5 Department of Laboratory Medicine, First Teaching Hospital of Tianjin University of Traditional Chinese Medicine, Tianjin, China; 6 Institute of Acupuncture and Moxibustion, First Teaching Hospital of Tianjin University of Traditional Chinese Medicine, Tianjin, China; 7 Department of Oncology, First Teaching Hospital of Tianjin University of Traditional Chinese Medicine, Tianjin, China

**Keywords:** immunity, metabolism, microbial metabolites, microbiota, precision medicine, stroke-associated pneumonia

## Abstract

Stroke-associated pneumonia (SAP) is the most common infectious complication following acute stroke. The limited efficacy of conventional antimicrobial therapy suggests that SAP may be fundamentally a syndrome driven by dysregulated cross-system interactions. This review proposes the “gut microbiota–immune axis” (GMIA) as a comprehensive framework for the development of SAP and systematically discusses the potential mechanisms by which post-stroke microbial-derived metabolic signals—including short-chain fatty acids (SCFAs), bile acids, tryptophan metabolites, and endotoxins—drive systemic immune reprogramming, predisposing patients to SAP. Based on the GMIA, we highlight several promising intervention strategies, including dietary modulation, precision antibiotic use, probiotics, fecal microbiota transplantation (FMT), supplementation with microbial metabolites, and receptor-targeted therapies, and summarize the current clinical translation related to the GMIA. Future research directions require high-quality clinical trials that integrate multi-omics data from the microbiome with immune biomarkers and clinical parameters. Such an approach is essential for constructing validated risk stratification models and advancing the management of SAP from empirical anti-infective treatment toward a precision medicine model centered on GMIA-based immune modulation.

## 1 Introduction

Stroke-associated pneumonia (SAP) was first characterized by Hilker and colleagues in 2003 (1). In accordance with the 2015 definition formulated by the Pneumonia in Stroke Consensus Group (PISCES), SAP is defined as pneumonia occurring within seven days of stroke onset in patients not requiring mechanical ventilation (2). As the predominant infectious complication following acute stroke, SAP exhibits an incidence ranging from 10% to 30% and is associated with a greater than threefold increase in mortality risk (3–8). In response to this significant clinical challenge, management strategies have historically centered on antimicrobial therapy (9). Nevertheless, evidence from multiple randomised controlled trials indicates that prophylactic antibiotic administration does not significantly reduce the incidence of SAP nor improve long-term neurological outcomes (10, 11). This apparent “therapeutic paradox” suggests that the pathophysiology of SAP extends beyond a conventional infectious etiology, thereby highlighting the inherent limitations of a purely antimicrobial therapeutic paradigm (12–14).

In fact, SAP emerges as a consequence of a complex interplay between multiple pathological processes, encompassing neural injury, immune dysregulation, gut microbiota dysbiosis, and compromised pulmonary defense mechanisms. Previous research has provided several explanatory frameworks: the “neuro-immune axis,” driven by sympathetic hyperactivity and activation of the hypothalamic-pituitary-adrenal (HPA) axis, which emphasizes central nervous system-mediated suppression of systemic immunity (15–17); the “brain–gut–lung axis” (18–20), linked through vagal imbalance, intestinal barrier disruption, and pulmonary immune remodeling; and a focus on mechanical and environmental risk factors, such as dysphagia, aspiration, and iatrogenic exposure (21). Notably, irrespective of whether the pathogenic cascade originates from central inflammation, autonomic dysfunction, or aspiration-related mechanical risks, these pathways are consistently accompanied by a common phenomenon—significant gut microbiota dysbiosis and concomitant alterations in immune phenotype. In recent years, investigators have elucidated the intricate mechanisms underlying post-stroke susceptibility to infection from diverse perspectives. The concept of “stroke-induced immune suppression” (SIIS) centers on the neuro-immune axis, delineating how central signals—notably excessive sympathetic activation—lead to systemic immune cell exhaustion, thereby impairing anti-infective defenses (22). Concurrently, the “brain–gut–lung axis” theory posits that post-stroke intestinal barrier damage, bacterial translocation, and endotoxin release may serve as direct or indirect triggers for distal pulmonary infection (18, 23). This implies that the gut microbiota may not function merely as a passive bystander affected by post-stroke dyshomeostasis, but rather as an active participant.The “gut microbiota–immune axis” (GMIA) does not refute the aforementioned pathways, but rather unifies them within a coherent causal sequence: an acute stroke event initiates, via neuroendocrine stress and intestinal ischemia/reperfusion injury, alterations in intestinal barrier permeability and a pronounced dysbiosis of the gut microbiota in both composition and function (14, 24, 25). This dysbiosis subsequently precipitates fundamental shifts in the profile of microbiota-derived metabolites with immunomodulatory properties—such as short-chain fatty acids (SCFAs) and bile acids—constituting a state of altered metabolic signaling (26–28). These abnormal metabolic signals subsequently act on immune organs and cells throughout the host via the systemic circulation, ultimately leading to a significant increase in the lungs’ susceptibility to pathogens and playing a key role in the development of SAP (29, 30).

Therefore, this review will focus on SAP and construct its framework according to a logical progression: from clinical manifestations to intestinal alterations, then to changes in metabolic signaling, systemic immune reprogramming, and increased susceptibility to SAP, and finally to discussions on intervention strategies and clinical translation. The discussion will employ a tiered evidence framework: building upon SAP-related clinical evidence, supplemented by evidence from the field of stroke immunology, and integrating insights from *in vitro* and mechanistic models, with the aim of constructing a rigorous argument regarding the pathogenesis of SAP.

## 2 The dynamic evolution and potential mechanisms of the GMIA in SAP

Clinical studies have demonstrated that patients with SAP exhibit characteristic alterations, including a reduction in short-chain fatty acid (SCFA)-producing bacteria, enrichment of opportunistic pathogens, and elevated markers of intestinal barrier damage (14). Furthermore, incorporating gut microbiota features into established clinical risk scores enhances the predictive accuracy for SAP (31). Given that the GMIA represents a pivotal integrative pathway in SAP pathogenesis, a systematic delineation of its specific mechanisms during disease progression is of considerable importance for informing the future development of targeted clinical interventions.

### 2.1 Starting point: post-stroke gut dysbiosis and barrier damage

#### 2.1.1 Characteristics of microbial composition imbalance

At the phylum level, the gut microbiome of stroke patients demonstrates an increased relative abundance of Firmicutes and Proteobacteria, concomitant with a significant reduction in Bacteroidetes (32). At the genus level, a marked depletion is observed in commensal bacteria responsible for SCFA production—such as *Faecalibacterium prausnitzii*, *Roseburia*, *Akkermansia muciniphila*, and *Bifidobacterium*spp. This depletion occurs alongside an enrichment of opportunistic pathogens, including *Enterococcus*spp. and members of the Enterobacteriaceae family (e.g., *Escherichia–Shigella*, *Klebsiella*, *Enterobacter*) (14, 33). These shifts in microbial taxonomy indicate a substantive breakdown of microbiome homeostasis and are associated with an enhanced pathogenic potential (34–36). Prospective cohort studies indicate that this specific microbial signature—characterized by reduced SCFA producers and expanded opportunistic pathogens—is significantly correlated with the risk of developing SAP and may serve as a potential predictive biomarker (31). Furthermore, a prospective clinical trial revealed that enterococci exacerbate systemic inflammation and promote SIIS in patients. This is mediated through an increase in interleukin-1 receptor antagonist (IL-1Ra) and a decrease in interferon-γ–induced protein 10 (IP-10) levels, thereby elevating the susceptibility to SAP (37).

#### 2.1.2 SNS-mediated intestinal barrier damage

Excessive activation of the sympathetic nervous system (SNS) following stroke constitutes a key mechanism underlying intestinal barrier disruption (22, 38). SNS hyperactivity directly induces apoptosis of intestinal epithelial cells, reduces goblet cell numbers and crypt proliferation, and compromises the integrity of the mucus layer (39). Concurrently, it downregulates intestinal Toll-like receptor 5 (TLR5) expression, thereby promoting the proliferation of flagellated bacteria (22). Furthermore, SNS overactivation may, via β-adrenergic receptor stimulation, drive a supraphysiological release of serotonin (5-hydroxytryptamine, 5-HT) and catecholamines. This can lead to impaired intestinal mucosal microcirculation and visceral vasoconstriction, theoretically contributing to intestinal ischemia–reperfusion injury (40). Although direct clinical imaging evidence of reduced mesenteric blood flow in stroke patients remains scarce, this pathophysiological sequence is supported by mechanistic rationale and animal studies. For instance, in the stroke-prone spontaneously hypertensive rat (SHRSP) model, sympatholytic agents have been shown to attenuate intestinal barrier damage (43). Corroborating this, clinical observations report elevated serum levels of intestinal injury markers, such as intestinal fatty acid–binding protein (I-FABP), in patients with acute stroke, indirectly suggesting the presence of intestinal ischemia/reperfusion pathology (41). Although there is correlative evidence supporting a sympathetic-mediated mechanism of barrier damage, its specific contribution to gut-to-lung bacterial translocation in the context of SAP requires further study and direct validation.

#### 2.1.3 Alterations in the gut microbial metabolome

The gut microbial metabolome undergoes profound remodeling following stroke. SCFAs, primarily acetate, propionate, and butyrate, constitute key immunomodulatory metabolites produced by gut microbiota through dietary fiber fermentation (42, 43). Fecal and plasma levels of total SCFAs, with notable reductions in butyrate and propionate, are decreased in patients with SAP (44–46). Clinical studies indicate that diminished intestinal SCFA levels in patients with acute ischemic stroke (AIS) correlate with mucosal immune dysregulation, and that this reduction may elevate the risk of bacterial translocation by compromising intestinal barrier integrity (47). Both clinical and preclinical evidence suggests that butyrate-producing commensals, such as *Prevotella fusiformis*, may confer protection against pneumonia via immunomodulatory mechanisms (48). Concurrently, abnormalities in secondary bile acid metabolism can further compromise the intestinal barrier, facilitating the systemic translocation of endotoxins (e.g., lipopolysaccharide, LPS) and the subsequent activation of a pro-inflammatory response (49, 50). Tryptophan metabolites, by modulating the T helper 17 (Th17)/regulatory T cell (Treg) balance, influence immune homeostasis; their dysregulation may contribute to an immunosuppressive state and heightened susceptibility to pneumonia (51, 52). Relevant animal studies have shown that tryptophanase inhibitors can reduce the formation of neutrophil extracellular traps (NETs) in stroke models, thereby improving lung injury (53, 54).

The interplay between the microbiota, the barrier, and metabolism following a stroke marks the starting point for the abnormal activation of the GMIA, laying the groundwork for subsequent systemic and pulmonary immune reprogramming.

### 2.2 Potential core metabolic signals

**Figure 1:**
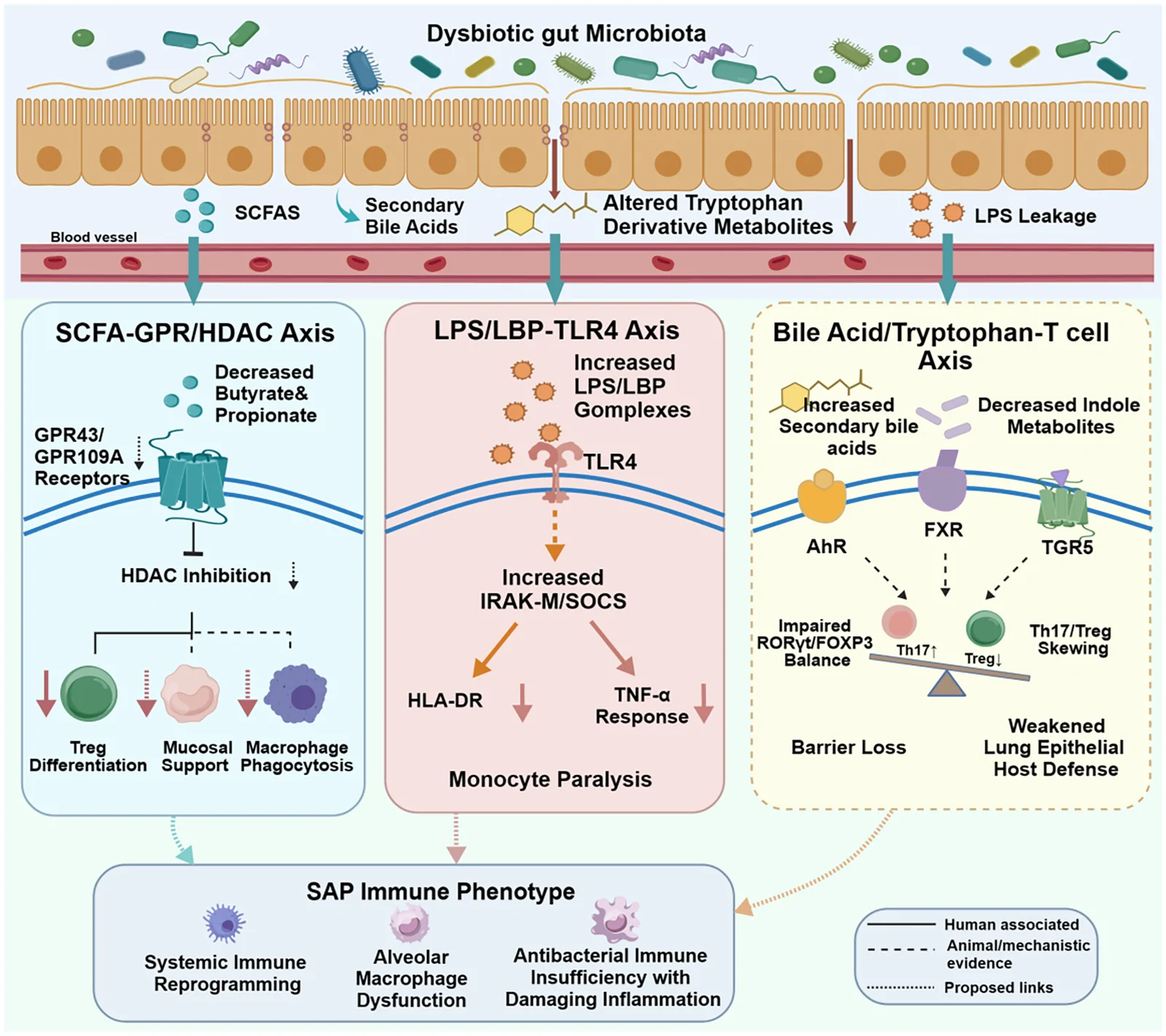
Potential metabolic-immune signalling pathways driving the GMIA. Suppressed SCFA signalling impairs Treg and macrophage functions; persistent LPS–TLR4 activation induces monocyte paralysis; and altered bile acid/tryptophan metabolism disrupts the Th17/Treg balance, all of which collectively compromise pulmonary host defence. Created with BioGDP.com (77).

Within the GMIA framework of SAP pathogenesis, the “core metabolic signals” currently attracting significant attention include signaling pathways involving SCFAs, bile acids, the tryptophan/tryptophan metabolism axis, and the LPS (55). These signaling pathways collectively point to an immune phenotype susceptible to SAP, characterized by systemic immune reprogramming that preferentially impairs antimicrobial clearance—most prominently reflected in reduced monocyte/macrophage reactivity and diminished antibacterial capacity of alveolar macrophages (AMs)—while distinct, tissue-resident or gut-homing–educated lymphoid compartments (e.g., γδ T17/ILC2/CD4^+^ Th17 cells and their downstream IL-17–driven recruitment) can remain engaged or even biased toward a pro-inflammatory phenotype within the lung interstitium and airway microenvironment. These two features can be understood as a temporally and spatially dissociated state: an early, predominantly systemic immunodeficiency lowers the threshold for pulmonary colonization (the “permissive window”), whereas locally sustained or ectopically primed inflammatory signaling—potentially amplified by microbial translocation, altered metabolite signaling, and neuro-immune efferent output—drives secondary tissue inflammation that compounds lung injury. In this framing, immunodeficiency and harmful inflammation act synergistically, not mutually exclusively, to shape SAP risk. Although some aspects are corroborated by clinical observations in stroke or SAP cohorts, others are primarily inferred from preclinical models and mechanistic studies.Consequently, for several of these pathways, their specific role within the proposed causal sequence of “post-stroke dysbiosis → metabolic alterations → immunosuppression → susceptibility to pulmonary infection” remains a working hypothesis awaiting comprehensive clinical validation (**Figure 1**).

#### 2.2.1 SCFA–GPR/HDAC axis: loss of homeostatic signaling

SCFAs—principally acetate, propionate, and butyrate—are key metabolic products derived from the microbial fermentation of dietary fiber. SCFAs exert immunomodulatory and barrier-protective effects via mechanisms including activation of G-protein–coupled receptors (e.g., G protein–coupled receptor 43 [GPR43], G protein–coupled receptor 109A [GPR109A]) and inhibition of histone deacetylase (HDAC) activity. These actions promote Treg differentiation, enhance intestinal mucosal barrier integrity, and sustain macrophage phagocytic capacity (56). Preclinical studies further demonstrate that gut-derived propionate can directly reprogram the immunometabolic state of AMs, thereby modulating their response to pulmonary injury and infection (57). Corroborating evidence from clinical research indicates that SCFAs, through GPR- and HDAC-mediated pathways, regulate T-cell homeostasis, mucosal barrier function, and pulmonary immunity in conditions such as childhood asthma (58, 59).

In the context of stroke, the pathophysiological relevance of the SCFA axis is underscored by the rapid onset of gut dysbiosis, barrier disruption, and bacterial translocation post-event, which are collectively associated with heightened infection susceptibility (56). A reduction in butyrate- and propionate-producing bacteria, leading to diminished systemic SCFA levels, may result in attenuated GPR43/GPR109A signaling and insufficient HDAC inhibition. This manifests as impaired Treg induction, compromised mucosal support, and reduced phagocytic/bactericidal capacity of macrophages. Furthermore, SCFAs are crucial for stimulating mucin secretion by goblet cells, thereby reinforcing the respiratory mucosal barrier; SCFA deficiency increases barrier permeability, facilitating pathogen invasion (57). Murine studies indicate that propionate, acting via GPR43, regulates macrophage metabolism to enhance bacterial clearance during pneumonia, whereas SCFA depletion diminishes the antibacterial efficiency of AMs (57).

Consequently, within the framework of SAP, a decline in SCFAs can be conceptualized as the concurrent loss of a critical metabolic signal that normally constrains excessive inflammation while preserving effective antimicrobial immunity in both the intestinal and pulmonary compartments. It is important to note, however, that direct causal evidence in humans linking specific alterations in the SCFA–GPR/HDAC axis to SAP pathogenesis remains limited; much of the current mechanistic understanding is derived from animal models and inferential studies.

#### 2.2.2 The LPS–TLR4 axis: monocyte paralysis and antimicrobial immune failure

Post-stroke disruption of the intestinal barrier and concomitant dysbiosis facilitate the sustained, low-dose translocation of enteric LPS into the systemic circulation. Within the circulation, LPS activate monocytes and macrophages via Toll-like receptor 4 (TLR4) signaling (60, 61). In contrast to the transient, pro-inflammatory activation induced by high-dose LPS during acute infection, chronic low-dose LPS exposure following stroke predisposes immune cells toward a state of “paralysis” or tolerance. Mechanistic studies indicate that upon activation of the LPS–TLR4 axis under these conditions, the canonical pro-inflammatory nuclear factor kappa-light-chain-enhancer of activated B cell (NF-κB) transcriptional program downstream of TLR4 is suppressed. Concurrently, negative regulatory molecules, such as interleukin-1 receptor–associated kinase M (IRAK-M), suppressor of cytokine signaling 1/3 (SOCS1, SOCS3), and A20, are up-regulated, and the anti-inflammatory interleukin-10 (IL-10)/signal transducer and activator of transcription 3 (STAT3) pathway is enhanced. This reprogramming ultimately results in a blunted release of pro-inflammatory cytokines (e.g., tumor necrosis factor-α (TNF-α), interleukin-6 (IL-6)), diminished antigen-presenting capacity, and reduced surface expression of human leukocyte antigen–DR (HLA-DR) (62, 63).

This mechanism aligns closely with the clinical phenomenon of SIIS. Observations in patients with acute stroke include reduced HLA-DR expression on peripheral blood mononuclear cells, attenuated pro-inflammatory cytokine production, and lymphocytopenia—immunosuppressive features that correlate with an increased risk of post-stroke infection (64). *In vitro* studies using human CD14^+^ monocytes exposed to LPS tolerance protocols recapitulate this phenotype, demonstrating decreased secretion of TNF-α alongside increased production of IL-10 (65).

Within the specific context of SAP, when these “tolerized” or “paralyzed” circulating monocytes and tissue-resident AMs encounter aspirated or inhaled pathogens, their suppressed TNF-α responsiveness, impaired antigen presentation, and compromised phagocytic capacity may be insufficient to mediate early bacterial clearance. This failure in initial host defense may permit bacterial colonization, proliferation, and the subsequent establishment of SAP.

#### 2.2.3 Bile acid/tryptophan–T-cell axis: immune polarization imbalance and pulmonary epithelial defense

The gut microbiota contributes to host immunoregulation through the metabolism of primary bile acids (e.g., cholic acid) into secondary bile acids (e.g., deoxycholic acid [DCA], ursodeoxycholic acid [UDCA]) and the conversion of dietary tryptophan into indole derivatives (e.g., indole-3-acetic acid [IAA]). Certain secondary bile acids, such as 3-oxolithocholic acid (3-oxoLCA) and isoallolithocholic acid (isoalloLCA), exhibit highly specific immunomodulatory functions: preclinical evidence indicates that 3-oxoLCA directly binds to and inhibits the transcription factor retinoic acid–related orphan receptor γt (RORγt) (critical for Th17 cell differentiation), whereas isoalloLCA enhances the expression of forkhead box P3 (FOXP3), the master regulator of regulatory T (Treg) cells, thereby finely tuning the Th17/Treg balance (45, 66, 67). Bile acid receptors, including the farnesoid X receptor (FXR) and Takeda G protein–coupled receptor 5 (TGR5), are expressed on intestinal epithelial cells and various immune cells. Signaling through these receptors regulates mucosal barrier integrity, cytokine production, and the generation of tolerogenic immune cell subsets (68–72). Preclinical studies suggest that FXR agonism can confer anti-inflammatory and protective effects in lung tissue, highlighting the potential for this axis to modulate the GMIA (73).

Concurrently, gut microbiota-derived tryptophan metabolites, such as indoles, activate the aryl hydrocarbon receptor (AhR), playing a key role in maintaining the Th17/Treg equilibrium and mucosal barrier homeostasis (74). Clinical studies in AIS patients show enhanced catabolism of tryptophan along the kynurenine pathway, with elevated levels of kynurenine and its downstream metabolite quinolinic acid correlating with infarct volume, stroke severity, and mortality risk (75). Mechanistic reviews indicate that these tryptophan metabolites, via AhR activation, can inhibit effector T-cell proliferation and promote an immune-tolerant state (52, 76). Therefore, stroke-induced alterations in tryptophan metabolism are poised to contribute to a pulmonary microenvironment characterized by diminished antimicrobial responsiveness and enhanced immunosuppression—a state conducive to SAP development. Similar to the bile acid axis, direct evidence linking specific tryptophan metabolites to human SAP pathogenesis is still evolving, but their role in post-stroke immunopathology is increasingly recognized.

Collectively, the core metabolic signals within the SAP-associated GMIA do not operate in isolation. The alterations in SCFAs, LPS, bile acids, and tryptophan metabolites described above ultimately converge to establish a susceptible immune phenotype. The strongest current evidence pertains to the clinical association between post-stroke monocyte immunosuppression (e.g., reduced HLA-DR expression) and infection risk, supported by mechanistic data from animal models linking stroke-induced gut dysbiosis to increased infection susceptibility. In contrast, the direct causal roles of specific metabolites—such as SCFAs, secondary bile acids, and indole derivatives—in the human SAP cascade require further validation.

### 2.3 Multi-pathway synergistic effects: systemic immune reprogramming from the gut to the lung

#### 2.3.1 Intestinal bacterial translocation and SAP

The significance of “bacterial translocation” in the pathogenesis of SAP does not supplant the aspiration theory, but it does provide an additional key pathway linking the source of infection to host susceptibility following a stroke. A pivotal 2016 study posited that not all microorganisms detected in post-stroke infections originate from the oropharynx, noting that in affected stroke patients, the majority of identified bacteria were gut commensals (78). In the accompanying mouse experiments for this study, 16S rRNA sequencing further revealed that the bacterial communities found in the lungs following a stroke could be traced back to the host’s small intestine, and that stroke-induced increased intestinal barrier permeability occurred prior to the dissemination of bacteria to peripheral tissues (78).

This translocation resembles a “selective dissemination.” Studies in aged stroke models demonstrate that while intestinal permeability increases and bacterial translocation occurs in both young and aged mice post-stroke, the profile of disseminated bacteria differs: *Escherichia*species are more frequently isolated in young mice, whereas *Enterobacter*species predominate in their aged counterparts (79). Further research indicates that aged stroke mice are more susceptible to spontaneous pulmonary bacterial infections, a susceptibility associated with more pronounced colitis and compromised mucosal barrier integrity (80). Thus, the post-stroke gut evolves toward a state that favors enteric-derived pulmonary colonization, a process modulated by age, barrier integrity, and host immune competence.

Current clinical evidence primarily supports a correlative relationship. Two recent prospective studies in patients with AIS have demonstrated a significant association between gut microbiota composition and SAP risk. One reported that a post-stroke reduction in SCFA-producing bacteria is linked to SAP and can improve the discriminatory power of conventional predictive scores (14). Another found that distinct gut microbiota profiles were associated with both SAP incidence and mortality risk, and integrating clinical indicators with gut microbiota and blood metabolite data further enhanced predictive accuracy (81). These findings collectively indicate a reproducible association between gut dysbiosis and SAP in humans. However, they substantiate the concept that “gut status influences SAP risk” and do not constitute direct clinical evidence that “bacteria translocate from gut to lung to cause pneumonia”.

This field is not without contention. A 2023 study noted that while stroke disrupts neuro-immune-metabolic homeostasis and promotes gut commensal overgrowth, this “gut microbiota overgrowth” alone is insufficient to explain Enterobacteriaceae colonization in the post-stroke lung. In other words, the observation of post-stroke dysbiosis does not automatically imply that pneumonia is directly caused by “ascending gut bacteria” (24). This underscores that SAP likely results from multiple interacting factors: stroke-induced intestinal barrier disruption facilitates egress of enteric bacteria; concurrent immunosuppression and pulmonary defense failure allow their persistence; and clinical factors such as aspiration, oropharyngeal microbiome imbalance, and mechanical ventilation may synergize with enteric dissemination to drive SAP pathogenesis.

Synthesizing existing research, a preliminary consensus is emerging: bacterial translocation is a key mechanism and risk amplifier in SAP development, but is not the sole or inevitable pathway (80).

#### 2.3.2 “Homologous homing” and phenotypic conversion of immune cells

The terms “homologous homing” and “phenotypic conversion” describe a process whereby the intestinal microenvironment first imprints a tissue-specific signature on immune cells, which are subsequently disseminated systemically, carrying this intestinal phenotypic information to distant sites such as the lungs. The local intestinal niche continuously shapes the differentiation and functional commitment of immune cells (82). The gut microbiota can drive the migration of group 2 innate lymphoid cells (ILC2s) from the intestine to the lung, a process modulated by the Proteobacteria–interleukin-33 (IL-33) axis (83). Thus, the gut functions not only as a source of metabolites but also as an immunological “training ground,” capable of exporting pre-programmed immune cells to influence the immune landscape of remote organs.

A 2023 study using a middle cerebral artery occlusion (MCAO) mouse model demonstrated that social isolation exacerbated brain and lung injury. This effect was partially mediated by the migration of small intestine–derived inflammatory γδ T cells to the brain and lungs, a process accompanied by elevated interleukin-17A (IL-17A) levels; inhibiting this cellular migration alleviated the social isolation–induced exacerbation of SAP (84). Another study using a sepsis-induced acute lung injury model supported the feasibility of “gut-derived immune cell migration to the lung.” It showed that small intestine–resident memory γδT17 cells could migrate to the lungs and instigate IL-17A–driven inflammation, a process facilitated by Wnt–CCL1 signaling in AMs (85). Corroborating this, in a murine SAP model, inhibition of CD147 reduced IL-17A expression in pulmonary γδ T cells while concurrently enhancing interferon-γ (IFN-γ) expression in pulmonary NK1.1^+^ and CD4^+^ T cells and reducing bacterial load (86). Collectively, this evidence suggests that post-stroke “gut-to-lung” immune reprogramming involves more than the simple translocation of cells. It may entail the systemic dissemination of immune cell populations that have been “educated” within the gut toward an IL-17–biased, pro-inflammatory, or mucosally dysregulated phenotype. This process disrupts a critical antimicrobial balance in SAP—namely, the equilibrium between an adequate, IFN-γ–mediated clearance response and a non-excessive, IL-17–mediated inflammatory response that can cause tissue injury.

#### 2.3.3 Systemic reprogramming of AMs

AMs are crucial resident phagocytes within the alveolar space, forming the respiratory tract’s primary defense against opportunistic pathogens. Under homeostatic conditions, they exhibit a degree of intrinsic hyporesponsiveness to prevent excessive inflammation from disrupting gas exchange. This physiological imperative for “restrained vigilance” renders AMs particularly susceptible to modulation by systemic neuro-immune signals following stroke (87).

A direct manifestation of this reprogramming is a decline in the phagocytic and bactericidal capacity of AMs. In a rat model of focal ischemic stroke, the post-stroke lung displays increased injury and inflammation, concomitant with reduced AM phagocytic activity and elevated IL-6 levels in pulmonary macrophages and endothelial cells. This suggests that systemic inflammation contributes to AM dysfunction (88). Placing this within a broader immunological context clarifies the underlying mechanism: AMs are highly plastic cells whose receptor repertoire, metabolic state, and transcriptomic profile can be durably remodeled by prior pulmonary insults (89). Similarly, the gut microbiota can distally “instruct” resident AMs; for instance, murine studies demonstrate that segmented filamentous bacteria (SFB) can reshape AMs, enhancing their resilience against exhaustion and functional collapse during respiratory viral infection (90). Applied to SAP, it is plausible that post-stroke neuroendocrine stress, systemic inflammation, and gut microbiome alterations do not act in isolation but collectively shift AMs from a state of “efficient patrolling, phagocytosis, and alarm” toward a new equilibrium characterized by “hyporesponsiveness, impaired clearance, and susceptibility to tolerance” (91).

Notably, this systemic AM reprogramming is not readily reversed by a single cytokine. In murine SAP models, intratracheal administration of interferon-γ (IFN-γ) yielded only limited improvements in pulmonary cytokine profiles, failed to prevent spontaneous infection, and even mildly impaired the clearance of aspirated *Streptococcus pneumoniae* (92). This indicates that AM abnormalities in SAP cannot be reduced to a simple “IFN-γ deficiency” or a static M1/M2 polarization state. Rather, they represent a profound functional reset, triggered systemically by the stroke event and subsequently amplified by local signals from the gut and lung compartments.

Beyond AMs, the maintenance of a pulmonary-susceptible state likely involves a cascade of reprogramming events affecting monocytes, dendritic cells, γδ T cells, T helper 17 (Th17) cells, group 2 innate lymphoid cells (ILC2s), and neutrophils (84). Studies on the gut–lung axis demonstrate that the gut microbiota can drive ILC2 migration to the lungs and pre-program the anti-infective function of AMs (90). Although not all these mechanisms have been directly validated in SAP, they provide a compelling framework for explaining the complex post-stroke pulmonary phenotype, which features concurrent local pro-inflammatory cell influx, hyporesponsiveness of resident myeloid cells, and the ultimate coexistence of failed bacterial clearance and lung tissue injury.

**Figure 2:**
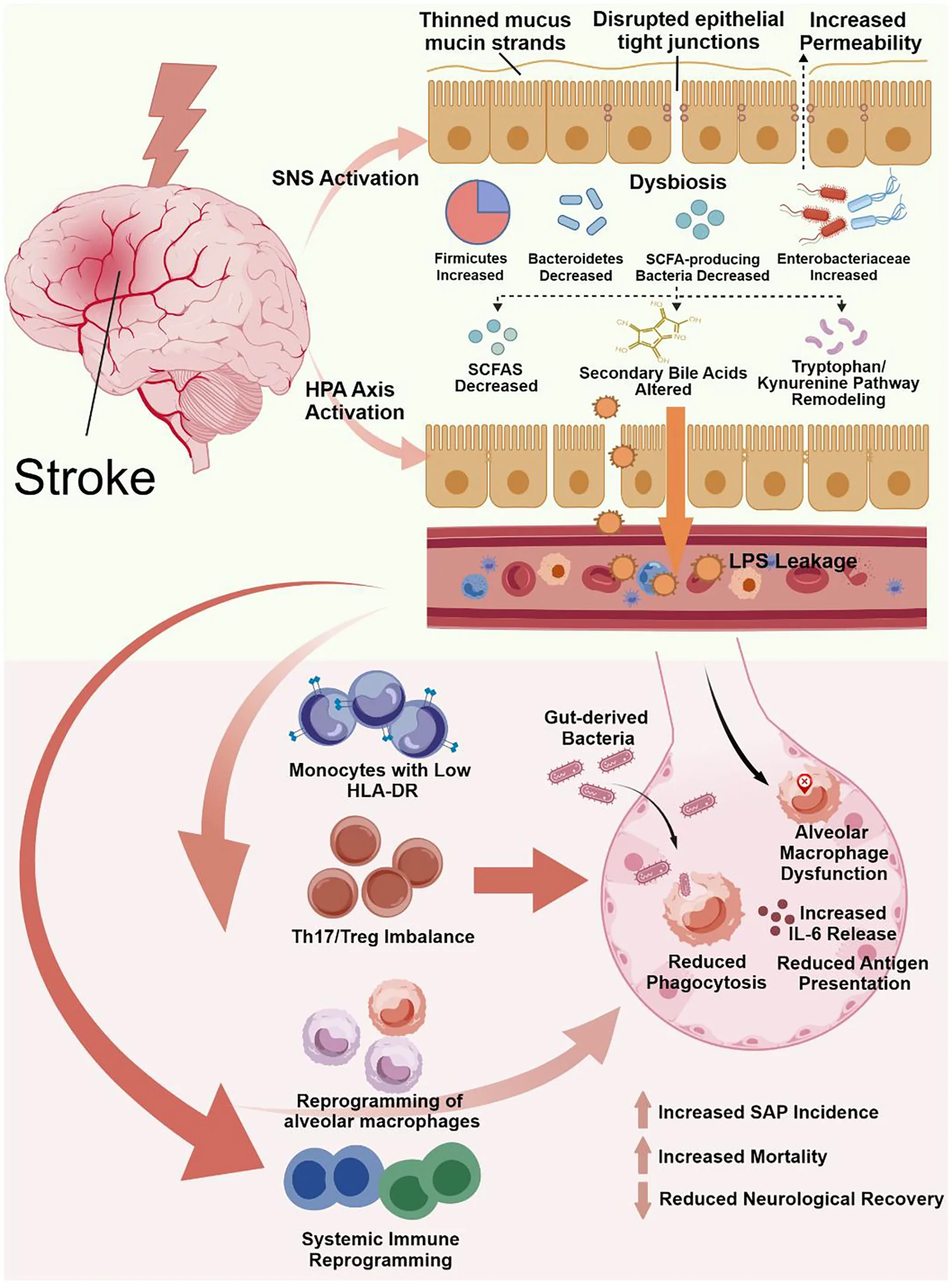
The integrated cascade of GMIA disruption promoting SAP. Acute stroke-induced neural activation disrupts the gut barrier and microbiota, driving systemic immunosuppression and impaired alveolar macrophage function, which together promote susceptibility to stroke-associated pneumonia. Created with BioGDP.com (77).

Importantly, GMIA framework does not imply that all pulmonary immune compartments are uniformly “hyperactive” or uniformly “paralysed” after stroke. In the early stages of SAP, the response is primarily characterized by local and systemic pro-inflammatory reactions (Th17-biased). Subsequently, under sustained stimulation, the body develops a compensatory anti-inflammatory syndrome, manifested as systemic immunosuppression (such as LPS tolerance in monocytes); however, at this stage, the lungs remain in a “mixed” immune state because the local inflammation has not subsided. The IL-17–biased, pro-inflammatory signal in the lung is primarily carried by tissue-resident or gut-imprinted lymphoid populations (γδ T17 cells, Th17/Th17-like CD4^+^ cells, ILC2-derived cascades) and their downstream neutrophil-recruiting chemokine milieu, whereas the critical defect in antimicrobial clearance maps onto the innate phagocytic—above all AMs (and, systemically, monocytes)—whose pathogen-sensing and bactericidal execution capacity is blunted by stroke-induced neuro-immune signaling, LPS/TLR4 negative feedback, and altered metabolite (e.g., SCFA) support. Thus, SAP can arise precisely because local inflammatory output and tissue injury are maintained (or even amplified), while the effector arm that clears bacteria is compromised—a dissociation that reconciles the “immunodeficiency vs. harmful inflammation” tension. It is important to emphasize that the strongest evidence for GMIA-mediated “gut-to-lung immune reprogramming” in SAP currently derives from preclinical models (**Figure 2**).

## 3 Intervention strategies targeting the GMIA and future prospects

Prior research has established that the post-stroke microbiome–metabolome profile is associated with the development of SAP and can enhance the predictive accuracy for SAP risk (14, 33, 93). Building upon this, several recent studies have identified dietary modulation, optimized antibiotic use, microbiome-based preparations, FMT, and the targeting of microbial metabolites as potential therapeutic avenues, positioning the gut microbiota as a key target for stroke management. While the theoretical rationale is increasingly clear, robust evidence of clinical efficacy requires further validation.

### 3.1 Dietary intervention: reshaping the GMIA at the nutritional level

Diet is the primary modifiable factor influencing the gut microbiota. Fiber-rich diets with polyphenols and unsaturated fats correlate with lower stroke risk and better outcomes, whereas high-calorie diets rich in saturated fat, sugar, and choline/L-carnitine are linked to higher stroke incidence and poorer prognosis (44, 94, 95). Fermentable dietary fiber improves gut dysbiosis, and its metabolites, SCFAs, provide dual immunomodulation: they reduce neuroinflammation via NF-κB inhibition and promote Treg differentiation through GPR43 activation, enhancing pulmonary immunity (96–98). In aged stroke mice, fecal transplants from young donors raised gut butyrate and lung VEGF, reducing pneumonia incidence (99). A randomized controlled trial in septic intensive care unit (ICU) patients indicated that supplementing enteral nutrition (EN) with soluble fiber increased the abundance of SCFA-producing bacteria and fecal SCFA concentrations, potentially reducing infection-related complications (100).

In practice, stroke patients frequently present with dysphagia and nutritional deficits, necessitating early EN. Some studies employing a combined strategy of “enhanced nutritional support plus probiotics” report improved nutritional status, reduced infectious complications, and better prognosis, suggesting that tailored EN formulations incorporating “SCFA-promoting” components may be a viable intervention (101).

From a GMIA perspective, dietary intervention in acute stroke should prioritize supporting SCFA-producing commensal bacteria by increasing fermentable fiber and reducing saturated fats and refined sugars, not merely calories (102). Optimal implementation requires validation in clinical trials and adherence to nutritional guidelines post-swallowing assessment.

### 3.2 Antibiotic strategies: from broad-spectrum prophylaxis to targeted adjustment

Prophylactic antibiotics do not reduce SAP incidence, functional outcomes, or mortality, and increase the risk of resistant bacteria and *C. difficile*infection (91, 103, 104). From a GMIA perspective, they deplete beneficial SCFA-producing bacteria, impairing barrier function and Treg differentiation, while promoting resistant Gram-negative colonization and endotoxin-driven TLR4 activation, exacerbating SIIS (32, 87, 105). Consequently, targeted therapy based on confirmed infection and susceptibility is advised (106).

Selective decontamination of the digestive tract (SDD) reduces ventilator-associated pneumonia in ICU patients, but its role in non-ventilated stroke patients is unproven, with concerns about resistance selection and impact on the stroke-perturbed microbiota (107–109). A 2025 survey found SDD is not routine practice in Australasian ICUs (110).

Potential optimizations include narrow-spectrum or topical SDD regimens for high-risk patients, coupled with microbiome monitoring, and combining necessary antibiotics with post-treatment microbiome restoration (e.g., probiotics or FMT) to shorten the immunocompromised window (111). As evidence for these strategies in SAP is lacking, they should be used cautiously and evaluated within clinical trials.

### 3.3 Microbiome modulators: probiotics, prebiotics, synbiotics, and postbiotics

Probiotics (e.g., *Lactobacillus*, *Bifidobacterium*) can improve neurological function, reduce inflammation, and enhance barrier integrity in preclinical stroke models, with some clinical studies supporting their use (112). They support SCFA-producing commensals, enhance barrier function, and modulate Th17/Treg balance, countering post-stroke immune dysregulation linked to SAP (37, 113–116). Specific commensals like *Prevotella fusiformis*show anti-pneumonia potential (48), and lactobacilli are neuroprotective (117). Probiotics may also ameliorate SAP by correcting sphingolipid metabolism (118).

Prebiotics (e.g., inulin, FOS, GOS, resistant starch) elevate gut SCFAs, attenuate neuroinflammation, and may reduce infection risk by limiting bacterial translocation and LPS burden in stroke models (94). Pueraria root resistant starch improves post-stroke intestinal barrier dysfunction, suggesting it may play a role in the prevention of pneumonia (119, 120).

Synbiotics (combined pro- and prebiotics) enhance microbial colonization stability and duration (121). They alleviate stress-related behaviors via ileal immune modulation dependent on the GMIA and are highly ranked for reducing infections, hospital stay, and antibiotic use, including ventilator-associated pneumonia (122–124), supporting their potential in SAP.

Postbiotics (inactivated microbes or their components, e.g., SCFAs, cell walls) offer immunomodulation without live-bacteria risks. Preclinical data show they can modulate immune cell function via GPCRs/TLRs and improve pulmonary infection outcomes (78, 125, 126), representing a potentially safer option for immunocompromised SAP patients, though evidence is preliminary.

In summary, these microbiome-based tools offer a relatively safe means to modulate the GMIA. We look forward to validation through large-scale clinical trials using standardized microbial strains, formulations, and dosing regimens.

### 3.4 FMT: a “strong intervention” to reset the gut ecosystem

FMT involves the transfer of processed stool or defined microbial consortia from a healthy donor to a recipient’s gastrointestinal tract, thereby achieving a “global reset” of the gut microbiota. It is under investigation for treating various conditions associated with dysbiosis, including inflammatory bowel disease, Parkinson’s disease, and stroke (127–129), and its efficacy is well established for recurrent *Clostridioides difficile*infection (130).

Current animal studies suggest that FMT may improve the pulmonary immune microenvironment and reduce susceptibility to infection by acting at an “upstream” level, through the rapid restoration of SCFA-producing bacteria, a reduction in the burden of drug-resistant opportunistic pathogens, the repair of the intestinal barrier, and a reduction in LPS exposure (127, 131, 132). Recently, FMT has been explored in stroke research. Animal studies show that aged stroke mice receiving FMT from young donors exhibit reduced behavioral deficits and neuroinflammation, correlated with elevated levels of SCFA-producing bacteria (e.g., *Akkermansia*) in the transplanted microbiota (129, 133). In the field of pulmonary infection, a case report describes the successful use of FMT to treat severe pneumonia with extensively drug-resistant *Klebsiella pneumoniae*, significantly improving the microbial profile and clinical outcome after antimicrobial therapy had failed (134), providing a pertinent proof-of-concept for SAP.

However, FMT in acute stroke carries risks: potential bacteremia/sepsis in immunocompromised patients (135), lack of standardized donor screening/preparation/administration (136), and unclear long-term ecological and theoretical carcinogenic risks, though direct cancer evidence in humans is scant (137, 138).

Thus, FMT should be considered a research strategy under strict oversight, not routine SAP care. Future work should trial FMT in the highest-risk SAP patients, identified via integrated microbiome–clinical scores, within controlled studies.

### 3.5 Supplementation of microbial metabolites and receptor targeting: precision modulation of immunity via “downstream signals”

#### 3.5.1 SCFA supplementation and signal modulation

SCFAs constitute one of the most central metabolic outputs of the GMIA. Studies related to respiratory diseases indicate that SCFAs not only directly inhibit the virulence and growth of various pathogens but also enhance the phagocytic and lysosomal fusion capacity of AMs, modulate neutrophil recruitment, and improve the inflammatory cytokine profile (42, 139–141). These effects collectively enhance host defense against bacterial and viral pulmonary infections. In the context of stroke and SAP, reduced plasma and fecal SCFA levels serve as markers of gut dysbiosis and barrier dysfunction and are associated with both SAP incidence and an immunosuppressive phenotype (87, 113). Therefore, supplementing SCFAs or their precursors is considered a “metabolic replacement” strategy aimed at correcting this deficit.

Specific strategies under investigation include: the oral or enteral administration of SCFA salts or their sustained-release formulations to elevate luminal and systemic SCFA concentrations (142); nutritional regimens rich in fermentable fiber and prebiotics to promote endogenous SCFA production via “precursor supply” (141); and the development of SCFA derivatives with improved pharmacokinetic profiles and tissue targeting for modulating local pulmonary immunity (26). While these approaches have demonstrated protective effects in models of pulmonary infection and chronic respiratory disease, their efficacy requires further validation in human studies with SAP as a primary endpoint.

#### 3.5.2 Bile acids, tryptophan metabolites, and their receptor-targeted modulation

Microbiota-derived metabolites of bile acids and tryptophan serve as key remote signaling mediators of the gut–lung axis. By activating specific receptors on pulmonary immune cells, they directly modulate the pulmonary immune microenvironment, potentially representing a critical mechanism influencing susceptibility to SAP. In infection models, unconjugated bile acids (e.g., lithocholic acid) generated by bacterial bile salt hydrolase (BSH) activity can inhibit coronavirus infection, highlighting a crucial role for microbial bile acid metabolism in immune defense (143). In primary sclerosing cholangitis–inflammatory bowel disease (PSC-IBD), strategies targeting bile acid metabolism and Th17 responses have shown therapeutic promise (144).

The gut microbiota forms an integrated “microbiota–metabolite–receptor” network via bile acid and tryptophan metabolism, converging signals through FXR/TGR5, AhR, and other pathways. This network plays a central role in maintaining mucosal barriers, regulating immune equilibrium, and attenuating neuroinflammation. Targeted modulation and clinical development of this network hold promise for improving the gut–lung immune axis via “metabotypic immunomodulation”.

#### 3.5.3 Postbiotics and engineered microbial strains

Compared to preparations containing live microorganisms, the direct use of “postbiotics” and engineered microbial strains offers potential advantages in terms of controllability and safety (145, 146).

Postbiotics—such as purified SCFAs, short peptides, or cell wall fragments—retain the capacity to inhibit pathogens, enhance intestinal barrier function, and modulate host immunity, presenting a promising alternative to live bacteria. Butyrate, a prototypical SCFA, serves as a classic postbiotic component. A murine model of bacterial pneumonia demonstrated that butyrate-producing commensals confer protection against pneumonia via the production of this microbial metabolite (48). Clinical research indicates that postbiotics can ameliorate sepsis-induced gut dysbiosis and associated acute lung injury (SALI) without the safety concerns inherent to live biotherapeutic products (147).

Engineered microbial strains involve the genetic modification of safe chassis bacteria (e.g., *Lactobacillus*strains engineered to produce butyrate or interleukin-10). Their advantages include targeted immunomodulation, controlled metabolite delivery, and enhanced safety profiles, as risks can be mitigated by deleting virulence genes or incorporating inducible expression systems (148). For example, engineered lung-adapted commensals (e.g., modified lactobacilli) can induce IL-17A secretion, enhancing the antibacterial function of γδ T cells and significantly reducing pneumonia susceptibility following antibiotic treatment (149). Such strains can constitutively or inductibly release anti-inflammatory molecules (e.g., IL-10) or reparative metabolites (e.g., butyrate), thereby mitigating inflammation while preserving antimicrobial capacity (148, 150), allowing for precise modulation of the pulmonary immune microenvironment.

From the perspective of GMIA, there is considerable potential for future precision interventions targeting the SCFA-GPR axis, the LPS-TLR4 axis, or the bile acid-FXR/TGR5 axis using “engineered microecological drugs”.

### 3.6 Other related strategies and prospects for integrated intervention

Beyond the direct microbiota- or metabolite-centered interventions described above, strategies that modulate host immunity and neuroendocrine pathways may also indirectly influence the GMIA. Traditional Chinese herbal medicines and complex formulations have demonstrated effects on gut microbiota composition, microbial metabolite profiles, and the GMIA in various models of stroke and brain injury. Their proposed mechanisms include improving gut microbial ecology by reducing the over-proliferation of potential pathogens (e.g., Enterobacteriaceae) and modulating gastrointestinal hormone secretion, thereby indirectly influencing disease progression (151). Host-directed immunotherapy strategies targeting post-stroke immunosuppression primarily focus on correcting key immune cell dysfunction and cytokine imbalance. By ameliorating T cell exhaustion and monocyte/macrophage paralysis, these approaches hold potential for complementary use with microbiome-based interventions (91, 152–154). Preclinical evidence suggests that combined targeting of innate (e.g., inhibiting dysregulated neutrophil migration) and adaptive immunity (e.g., restoring T cell function) may be more effective in preventing stroke-associated infections (152, 155). Beta-adrenergic blockers have been shown in animal models to partially reverse sympathetic-driven immunosuppression; large-scale clinical research in this area remains active (156).

**Figure 3:**
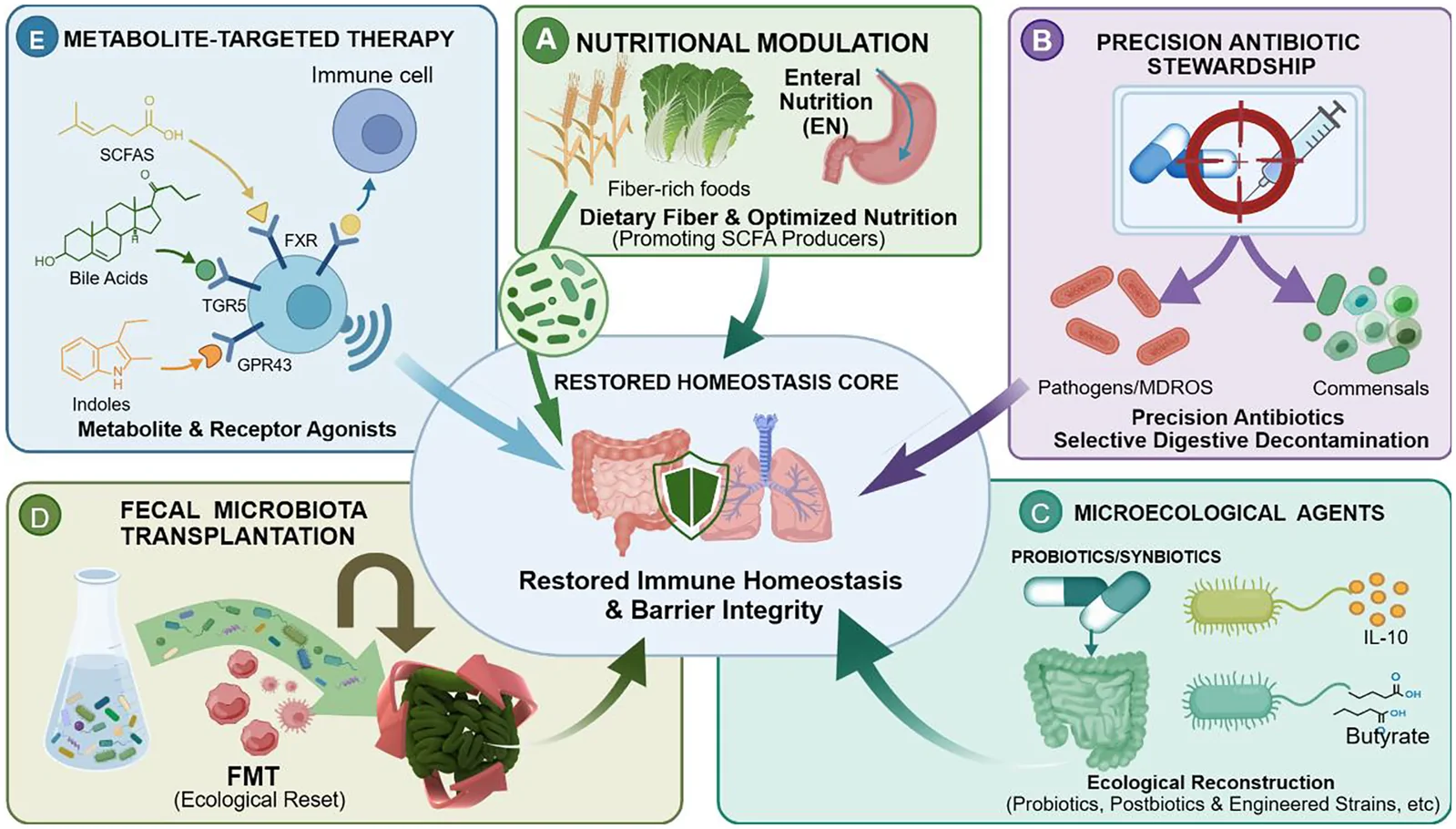
GMIA‑targeted intervention strategies for SAP. This figure illustrates core GMIA‑targeted strategies—encompassing **(A)** Nutritional Modulation; **(B)** Precision Antibiotic Stewardship; **(C)** Microecological Agents; **(D)** Fecal Microbiota Transplantation; **(E)** Metabolite‑Targeted Therapy. All strategies converge to restore gut barrier integrity and pulmonary‑intestinal immune homeostasis, thereby lowering the risk of SAP. Created with BioGDP.com (77).

In summary, future GMIA-targeted SAP interventions should move from single-agent to integrated strategies. This involves first developing risk models using gut microbiota and metabolic profiles to identify a “high-risk metabotype”. A tiered plan can then combine dietary optimization, microbiome modulators, and standard nutrition. For high-risk cases, intensive options such as FMT or metabolite supplementation may be considered (**Figure 3**).

## 4 Clinical translation in SAP

### 4.1 Validated risk stratification tools and biomarkers for SAP

The diagnosis of SAP currently relies primarily on clinical presentation, radiological evidence, and microbiological assessment, with blood biomarkers more commonly employed for early risk identification, aiding the differentiation of bacterial infection, and guiding antimicrobial therapy (2). Among tools that have undergone clinical validation, the A2DS2, ISAN, and AIS-APS scores are the most widely applied SAP prediction models. Their core variables include age, atrial fibrillation, dysphagia, stroke severity, sex, and pre-stroke functional status. These scores serve as essential baseline risk-stratification tools in SAP translational research (157–159).

Regarding hematological inflammatory markers, C-reactive protein (CRP) ranks among the most frequently evaluated biomarkers in SAP research. An early post-admission elevation in CRP correlates with stroke severity, a heightened inflammatory response, and an increased subsequent risk of infection. However, its specificity is confounded by factors including brain tissue injury, the systemic stress response, and comorbid conditions (160). In contrast, procalcitonin (PCT) demonstrates greater specificity for bacterial infection. Consequently, it holds significant translational value for the early detection of SAP, the differentiation of bacterial pneumonia, and antimicrobial stewardship. Its diagnostic performance, nonetheless, is typically interpreted in conjunction with clinical assessment scores, radiological findings, and microbiological evidence (161). Measures such as the total white blood cell count, neutrophil percentage, and the neutrophil-to-lymphocyte ratio (NLR) reflect the systemic inflammatory and stress state following stroke and are associated with SAP risk and poor prognosis; however, these indices are readily influenced by stroke severity, corticosteroid administration, pre-existing infections, and other inflammatory pathologies (162).

SIIS constitutes a key mechanism in the pathogenesis of SAP. Peripheral lymphopenia, reduced monocyte human leukocyte antigen–DR (mHLA-DR) expression, elevated IL-10 levels, and impaired T cell function are all associated with heightened susceptibility to infection. Among these, mHLA-DR serves as one of the most representative indicators of post-stroke immune paralysis. Low mHLA-DR expression reflects diminished monocyte antigen-presenting capacity and a blunted endotoxin response (64). Moreover, the decline in mHLA-DR is correlated with an increased risk of post-stroke infection and mortality. Consequently, it is frequently incorporated into dynamic monitoring protocols—typically on days 1, 3, and 5—in studies investigating biomarkers for stroke-associated infections (163).

The elevation of typical pro-inflammatory mediators, IL-6 and TNF-α, shows a positive correlation with stroke severity, as assessed by the NIHSS score, and the risk of secondary infection (164). In contrast, the profile of IL-10 is more complex. Its levels are markedly increased in patients with SAP and correlate positively with the Pneumonia Severity Index (PSI). This elevation, however, does not represent a protective response, but rather reflects an exaggerated anti-inflammatory reaction that contributes to an immunosuppressive state (165). For clinical risk prediction, the IL-6/IL-10 ratio, either alone or in combination with the A2DS2 score, may be a more sensitive indicator of the pro-/anti-inflammatory imbalance and could provide early warning value for SAP risk.

Overall, the currently more established translational strategies for SAP do not rely on a single biomarker. Instead, they favor the integration of multiple parameters into predictive models, encompassing clinical risk scores, infection markers such as PCT or CRP, indicators of immunosuppression, and the dynamic progression of the clinical condition.

### 4.2 Microbiota-related biomarkers with emerging evidence

In recent years, gut microbiome-related biomarkers have garnered significant attention as emerging tools for predicting the risk of SAP. Patients with AIS frequently exhibit a reduction in gut microbiota diversity, a decrease in SCFA-producing bacteria, and an expansion of conditionally pathogenic bacteria. This “stroke-associated dysbiosis” is implicated in stroke severity, neurological functional outcomes, and infectious complications (44). Emerging evidence suggests that an increase in Enterobacteriaceae, Proteobacteria, or other facultative anaerobic opportunistic pathogens may reflect a shift in the gut ecological niche toward a pro-inflammatory and pathogen-permissive state, thereby elevating the risk of pulmonary or secondary infections (129). Conversely, a reduction in butyrate-producing bacteria and other strictly anaerobic commensals may compromise the intestinal epithelial barrier, the induction of Tregs, and pulmonary mucosal immune homeostasis, providing a biological rationale for the susceptibility to SAP mediated by GMIA disruption (166). Research focusing on the gut microbiota and its metabolic product SCFAs has identified specific taxonomic associations. For instance, SAP patients demonstrate a marked decrease in the beneficial genus *Roseburia*alongside an increase in opportunistic pathogens. Notably, *Roseburia*serves as an independent protective factor against SAP (14). Another study revealed a protective role for *Finegoldia*, while *Lactobacillus*was associated with an increased risk of SAP (33).

Intestinal barrier disruption, translocation of bacterial products, and LPS-associated inflammatory signaling pathways link gut microbiota alterations to systemic immune activation or immunosuppression. Consequently, markers of intestinal barrier function and microbial metabolites are also emerging as potential supplementary indicators for SAP risk assessment. However, a study involving 331 stroke patients demonstrated that while plasma levels of LBP and soluble CD14 (sCD14) were significantly higher in the SAP group than in the non-SAP group, multivariable analysis revealed limited practical value of LBP as a stand-alone risk marker for SAP (167), and its predictive utility requires further validation. In contrast, microbial metabolites such as SCFAs, bile acid derivatives, and tryptophan metabolites, which regulate myeloid cells, T cell responses, and pulmonary anti-infective immunity, are considered promising functional markers bridging gut dysbiosis to pneumonia susceptibility. The combination of gut microbiota profiling and circulating biomarkers, such as antibodies against the *N*-methyl-D-aspartate receptor (NMDAR) and butyrate, has been shown to significantly improve the accuracy of SAP prediction, with NMDAR exhibiting the highest standalone predictive performance (81). Meanwhile, TMAO and its associated microbial metabolic pathway are more closely linked to atherosclerosis and stroke risk rather than directly to post-stroke infectious complications, and evidence supporting its role as a specific predictive marker for SAP remains insufficient. I-FABP has been demonstrated in human studies to reflect intestinal mucosal injury in AIS and correlates with a state of post-stroke intestinal hyperpermeability (41), whereas diamine oxidase (DAO) is a more established marker of intestinal mucosal ischemia/injury, with a solid research foundation in intestinal ischemia (168). Nevertheless, both I-FABP and DAO have not yet undergone extensive validation in SAP-specific cohorts, and therefore they are currently best categorized as “candidate barrier injury markers”.

Currently, microbiota-related biomarkers for SAP remain in the discovery and external validation phases, with a lack of standardization regarding sampling timing, sequencing platforms, analytical pipelines, and clinical thresholds. Therefore, a more promising and potentially feasible direction for translating these biomarkers into routine clinical practice may lie in integrating fecal microbiota profiles, circulating immunosuppression markers, infection indicators such as PCT or CRP, and established clinical risk scores (e.g., A2DS2 or ISAN) into comprehensive predictive models. This multi-parameter approach could enhance both the robustness and interpretability of early SAP prediction.

### 4.3 Clinical trials of SAP related to the GMIA

Currently, SAP clinical trials related to the gut microbiome are still in a developmental stage, and formal cohorts directly targeting SAP remain limited. Xia et al., in their prospective work on acute ischemic stroke, further linked SAP to quantifiable microbial signatures: SAP patients exhibited depletion of *Roseburia*and enrichment of opportunistic pathogens. The microbial dysbiosis index (MDI) composed of these features was associated with approximately a twofold increase in SAP risk and corresponded with reduced fecal SCFA levels and elevated intestinal barrier markers (14). Additionally, a study on probiotic intervention demonstrated that, in the treatment of pneumonia in elderly patients during stroke recovery, the observation group receiving additional probiotics showed a significantly higher treatment response rate (95.5% vs. 80.0%) and a lower incidence of adverse reactions (2.3% vs. 20.0%) compared to the conventional treatment control group (169). In critical care medicine, existing research has established that probiotics or synbiotics can be used to prevent ventilator-associated pneumonia and other hospital-acquired infections, providing indirect support for gut microbiome–targeted interventions in SAP (170, 171). However, the unique pathophysiological background of stroke patients—including dysphagia, impaired consciousness, aspiration risk, and immunosuppression—requires further investigation to determine how these findings can be extrapolated.

While current interventional research for SAP prevention primarily focuses on prophylactic antibiotics, a GMIA perspective suggests that broad-spectrum antibiotics may simultaneously reduce potential pathogens and disrupt commensal microbiota (155). Consequently, future studies should concurrently evaluate infection outcomes, alterations in microbiota structure, antimicrobial resistance, and immune function.

## 5 Conclusions and future prospects

SAP is likely not a simple infection but the outcome of stroke-induced, GMIA-mediated systemic immune-metabolic dysregulation. This framework challenges purely antimicrobial strategies and supports a shift toward early risk stratification and multi-target interventions based on host immune-metabolic status. It is important to emphasize that while the significance of GMIA in the pathogenesis of SAP does not supersede the inhalation theory, it does provide a crucial additional pathway linking the source of infection to the host’s susceptibility following a stroke, and offers a new perspective for research into the pathogenesis of SAP.

Current human evidence primarily confirms associations between gut microbiota profiles and SAP risk; the full GMIA causal chain requires further validation. Future mechanistic work should employ multi-omics to define how specific post-stroke gut metabolites modulate pulmonary immune cells (e.g., alveolar macrophages, γδ T cells) and clarify the relative roles of aspiration versus gut-origin translocation.Clinically, future GMIA intervention trials should use stratified designs, enrolling high-risk patients identified by dysphagia, stroke severity, clinical scores, biomarkers, and dysbiosis signatures. Endpoints should extend beyond SAP incidence to include antibiotic use, resistant bacteria colonisation, microbiota/SCFA changes, immune phenotype restoration, hospital stay, and 90-day outcomes. Priorities include initiating large RCTs with SAP as the primary endpoint to test specific probiotics, fiber-enhanced nutrition, or metabolite supplements. Subsequent efforts should develop integrated prediction models combining clinical scores, infection/immune markers, and microbial features for early high-risk identification. In strictly defined, highest-risk cohorts, the feasibility of intensive interventions like FMT or engineered strains—with standardised donor screening and safety protocols—should be explored.

Ultimately, SAP management should evolve from reactive treatment to proactive susceptibility intervention. Through GMIA-informed risk stratification, personalised strategies combining microbial modulation, immune regulation, and targeted nutrition could be deployed in high-risk individuals. Integrating these approaches with established dysphagia management and early rehabilitation may reduce SAP incidence and improve stroke outcomes.
